# Trauma, dream, and psychic change in psychoanalyses: a dialog between psychoanalysis and the neurosciences

**DOI:** 10.3389/fnhum.2013.00877

**Published:** 2013-12-17

**Authors:** Tamara Fischmann, Michael O. Russ, Marianne Leuzinger-Bohleber

**Affiliations:** Sigmund-Freud-InstitutFrankfurt, Germany

**Keywords:** psychic trauma, dreams, psychoanalysis, neurosciences, EEG-fMRI

## Abstract

To many psychoanalysts dreams are a central source of knowledge of the unconscious—the specific research object of psychoanalysis. The dialog with the neurosciences, devoted to the testing of hypotheses on human behavior and neurophysiology with objective methods, has added to psychoanalytic conceptualizations on emotion, memory, sleep and dreams, conflict and trauma. To psychoanalysts as well as neuroscientists, the neurological basis of psychic functioning, particularly concerning trauma, is of special interest. In this article, an attempt is made to bridge the gap between psychoanalytic findings and neuroscientific findings on trauma. We then attempt to merge both approaches in one experimental study devoted to the investigation of the neurophysiological changes (fMRI) associated with psychoanalytic treatment in chronically depressed patients. We also report on an attempt to quantify psychoanalysis-induced transformation in the manifest content of dreams. To do so, we used two independent methods. First, dreams reported during the cure of chronic depressed analysands were assessed by the treating psychoanalyst. Second, dreams reported in an experimental context were analyzed by an independent evaluator using a standardized method to quantify changes in dream content (Moser method). Single cases are presented. Preliminary results suggest that psychoanalysis-induced transformation can be assessed in an objective way.

## Introduction

In 2006, declared as “the Year of Freud,” one could easily get the impression that the dialog between psychoanalysis and the neurosciences was the most important window that opened modern day psychoanalysis to the world of contemporary scientific discourse. Can we, as psychoanalysts, initiate a fruitful dialog with neuroscientists and gain additional knowledge of the unconscious, psychoanalysis' specific research object?

Throughout his entire life Freud had hoped that new developments in the neurosciences would contribute to exploring psychoanalytic processes from a natural scientific point of view. In many of his historical and theoretical papers the South African neuropsychologist and psychoanalyst Mark Solms substantiates that Freud—due to the standard of neuroscientific methods during his time—turned his back on this vision and defined psychoanalysis as a solely psychological science of the unconscious. Over the past few years, recent developments in the neurosciences, e.g., investigating the living brain with the help of neuroimaging techniques, as well as the neuro-anatomic method, as described by Solms and other psychoanalytic researchers, have stimulated and intensified the interdisciplinary dialog between psychoanalysis and the neurosciences. The first issue of the international journal “Neuro-Psychoanalysis” was printed in 1999, featuring well-known neuroscientists and psychoanalysts controversially discussing in detail topics such as emotion, memory, sleep and dreams, conflict and trauma, as well as conscious and unconscious problem-solving processes. The international Society for Neuro-Psychoanalysis was founded in the year 2000 contributing to an exchange between both scientific disciplines via regular congresses.

Apparently a growing number of worldwide research groups have begun to realize that the neurosciences and psychoanalysis can benefit from each other in interesting ways. By now the neurosciences are equipped with objective and precise methods of verifying hypotheses about human behavior, while psychoanalysis, based on its rich experience with patients and its unique method of field research has developed a variety of different models in order to conceptualize the multi-layered and complex observations derived from the psychoanalytic situation and to test them by means of its specific form of empirical research—clinical psychoanalytic research. Psychoanalysis' explanatory models and insights can conversely be of interest to neuroscientists and raise specific research questions (see e.g., Fischmann et al., 2012a; Ruby, 2013).

Sometimes also empirical studies evoke challenging research questions for both research fields. In the on-going LAC-Depressionstudy (see below), for example, one interesting and unexpected finding for both research fields is that a large majority of chronically depressed in long-term psychoanalytic therapy suffered from severe traumatization during childhood. The scientific discourses on the long-term effects of traumatizing experiences can be traced back to the mid 19th century (Sachsse et al., 1997; Bohleber, 2000b, 2010; Mertens and Waldvogel, 2008) when Freud developed his first theoretical understanding of trauma in 1895 in his “Project for a Scientific Psychology” (Freud, 1950[1895]). In the 1920s he developed the structural model of psychoanalysis, a “solely psychological” theory. Nevertheless, as is well known, Freud always kept his interest in the neurological basis of psychic functioning, particularly also concerning the topic of trauma.

After World War II the consequences of “man-made disasters” refocused the professional attention on trauma. For one, the extremely traumatizing experiences of the Holocaust, which led many survivors to reach out to psychoanalysts in the form of treatment or for an assessment due to reparation claims, compelled a reviewed analysis of the short- and long-term consequences of extreme traumatization. Moreover, the treatment of survivors' children conveyed the insight that traumatic experiences of this enormity also encroach on the lives of the following generations. “Man-made disasters” have various transgenerational effects, not only for the directly involved families, but also for society as a whole and for the trauma's representation within the collective memory and group identity, subjects for further interdisciplinary dialog (Bohleber, 2000a, p. 795, 2010)^1^. Many decades ago, Hans Keilson (1979), amongst others, characterized Auschwitz as a place “which our language cannot reach,” where the traumatic experience destroyed the human shield that is the structure of meaning. The traumatic experience carves itself into the body and directly influences the organic base of psychic functions. Psychic space and the ability to symbolize are destroyed (Laub et al., 1995; Bohleber, 2000b, 2010; Kogan, 2002). These findings from clinical psychoanalytic research have been pursued by many psychiatric and neurobiological researchers in the last years (see following section).

## Psychoanalytic and neurobiological trauma research

In any age traumatization can lead to severe incursions of a person's psychic structures (also see Leuzinger-Bohleber, 2010, 2013; Leuzinger-Bohleber et al., 2010). One of the effects of an acute, severe traumatization is that the affected person is abruptly seized from reality by the traumatic experience: within a dissociated condition he now experiences the reality surrounding him in a completely different way, unreal, fey, separated from all other people, isolated and lonely. Intuitively he realizes that this experience depicts an infraction in his life that he will carry within himself from now on. Nothing will be as it was before. Psychoanalysts know by treating severely traumatized patients that they did not find their way back into their old lives after such an experience: psychically they are “never totally present,” they have permanently lost their foothold, feel disconnected toward others and never regain the sense of being the active center of their own lives.

These psychoanalytic insights on the psychodynamics and genesis of traumatization are generally based on psychoanalysts' intense work with individual patients seeking relief from their psychic or psychosomatic problems. Most often the insights about unconscious determinants of psychic grief not only turn out to be “healing” pertaining to the physical symptoms but also in a meaning-giving way, in the sense that certain, until now, unknown effects of sustained traumatization are now recognized as memories or memorials of the personal, distinctive life story and psychically integrated.

In contrast psychiatric and neuroscientific literature debate trauma centered on “posttraumatic stress disorder.” The DSM-IV definition of posttraumatic stress disorder (PTSD) is regarded as the international standard and its definition has become the basis of many interdisciplinary studies. It must be taken into account that this definition is solely descriptive in nature, and does not give an account about which psychic and/or neurobiological mechanisms lie at the root of this psychic traumatization. In terms of the DSM-IV, posttraumatic stress disorder is “the development of characteristic symptoms after being exposed to a traumatic event.” This event is defined as: “The person has experienced, witnessed, or been confronted with an event or events that involve actual or threatened death or serious injury, or a threat to the physical integrity of oneself or others.” (DSM Criteria for PTSD). Such an event impacts the subject in the form of an external, massive stressor and changes the structural features which have been formed in part by genetics, prenatal and early childhood attachment, and experiences in the outside reality. This impact is identified as a threat by the brain and therefore quickly leads to a somatic stress reaction accompanied by severe psychic reactions (cf. Sachsse and Roth, 2008; Reinhold and Markowitsch, 2008).

Among others, DSM-IV lists the following symptoms for PTSD: intense fear, helplessness or horror, recurrent and intrusive distressing recollections of the event, persistent avoidance of stimuli associated with the trauma, as well as persistent symptoms of increased arousal. The causes for traumatising situations are e.g., wars, natural disasters, severe accidents, as well as harm caused by others such as torture or rape” (DSM-IV, p. 487).

What consequences do these different approaches have for the treatment of traumatized individuals? From a psychoanalytic point of view today, in cases of traumatic experiences the natural stimulus barrier is interrupted by unforeseen, extreme experiences, usually linked to a threat to life or mortal fear. The ego is exposed to an extreme feeling of powerlessness and inability to control or manage the situation and is therefore flooded with panic and extreme physiological reactions. The flooding of the ego leads to a psychic and physiological state of shock. The traumatic experience also destroys the empathic shield of the internalized primary object, the confidence in the constant presence of good objects, and the expectancy of human empathy. In trauma the inner, good object, the negotiator between self and surroundings becomes mute (Hoppe, 1962; Cohen, 1985). The feeling of continuity and the basic sense of one's own life are lost. Therefore the “narrative,” and the “meaning-giving” psychotherapeutic dimension are essential for treating the group of severely traumatized persons. At the same time psychiatric and neuroscientific findings on the brain function of traumatized patients may be relevant even for psychotherapists, as we try to illustrate in this paper.

Hence in our introduction we try to bridge the gap between psychoanalytic and neuroscientific findings on trauma. In the next section we apply this knowledge to an on-going study by simultaneously examining chronically depressed patients with EEG and fMRI during their psychoanalytic treatment. Our example of a single case study will illustrate in the last section how a change of manifest dream contents, as they are portrayed in psychoanalytic sessions, can be contrasted by changes in the contexts of dreams in the sleep laboratory. As we have discussed in previous works, within the transference relationship with the analyst when dealing with severely traumatized, depressive patients, it is inevitable that the traumatic experience is revived and thus understood in detail in its biographical (“historical”) dimension (Fischmann et al., 2012a; Leuzinger-Bohleber, 2013). Only then does trauma in its enclosed, psychic existence become accessible to therapeutic work: the unutterable horror is linked to visualizations, metaphors and eventually to verbalizations. Dreams are often helpful in this context: many analysands convey indicators for an incipient symbolization process and the conclusive onset of “meaning-giving” therapeutic coping with the traumatization. Therefore the changes of dreams during psychoanalysis with severely traumatized patients could indicate that a symbolization process of the trauma has taken place—and thus indicating significant transformations in the inner world of the patient. In this paper we would like to report on an attempt to measure such transformation processes in the manifest dreams of chronic depressed analysands not only by clinical psychoanalytic observations but also by a theory-guided content analysis of dreams developed by Moser and von Zeppelin (1996) that is accepted by the non-psychoanalytic academic community. In this model the state of the art of experimental and neurobiological dream/sleep research has been integrated.

## Dream and depression

In dream research dreaming is described as a thought-process in which our inner system is engaged in processing information (Dewan, 1970). Inner (cognitive) models are constantly being modified in coordination with what is perceived. In contrast to dreaming reactions to our environment are immediate during the waking state, thus enabling information consolidation into memory only limited by capacity restrictions of the system itself. Nevertheless, consolidation processes do continue during sleep in an “off-line” modus, thus enabling integration into the long-term memory here as well (Stickgold and Walker, 2007).

According to Moser and von Zeppelin^2^ (1996), both psychoanalysts and dream researchers, the so-called “dream complexes,” activated by current events, process the entirety of information deriving from unsolved conflicts and traumatic situations while dreaming. The dream searches for solutions or rather best possible adaptations for these dream complexes. A dream, which is usually pictorial, consists of at least one situation produced by a “dream-organizer.” Dream-organization may be considered—according to Moser—as a bundle of affective-cognitive procedures, generating a micro-world—the dream—and controlling its course of action. Within this system the “dream-complex” is a template facilitating dream organization.

**Figure 1:**
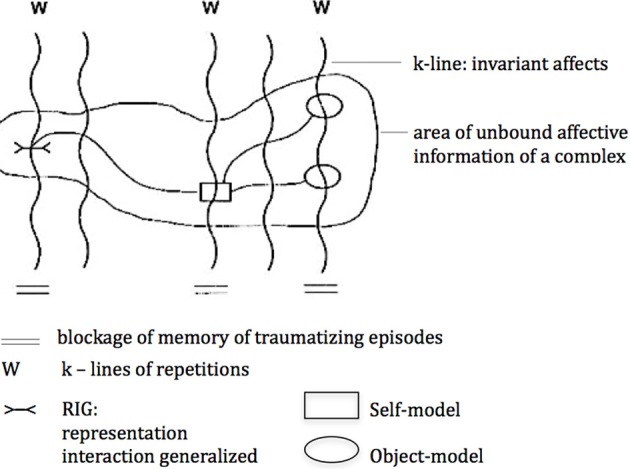
**Memory Model of conflictuous complexes according to Moser and von Zeppelin (1996)**.

Thus, it may be assumed that a “dream-complex” originates from one or more complexes stored in the long-term-memory, rooted in conflictuous and/or traumatizing experiences, which found their condensates in *introjects*. These introjects are closely related to triggering stimuli from the outside world and structurally similar to stored situations of the complex. The searched for solution of the complex is governed by the need for security and the wish for involvement, i.e., the *security-principle* and the *involvement-principle*, managing the dream-organization. Wishes within these complexes are links between self- and object-models and RIGs (i.e., Representation Interaction Generalized), which are accompanied by convictions and a hope for wish-fulfilment. Conflictuous complexes are areas of bundled wishes, RIGs and self- and object-models with a repetitive character, thus creating areas of unbound affective information. Affects within such an area are inter-connected by k-lines, which are blocked and therefore cannot be located. In order to solve these conflictuous complexes it is necessary to retrieve this affective information into a relational reality in order to make them come alive and locatable (cf. Figure 1). This is attempted in dreams, their function being the search for a solution of the complex. This search for a solution within a dream again is governed by the above-mentioned *security-principle* and *involvement-principle*. The following illustration may serve as an elucidation of this model.

## Measuring the outcomes of psychoanalytic treatment: an interdisciplinary challenge

Could the psychoanalytic “meaning-giving” transformation processes in the inner world of depressed patients—such as dreams—also become a part of studies based on the new possibilities of neuroimaging studies? Eric Kandel is convinced that psychoanalysis must apply these new methods in order to prove neurobiologically the sustainability of its results (Kandel, 2009 and verbal accounts) otherwise it will vanish from the world of science and only be remembered as a historical relic, a memory attesting to Sigmund Freud's enlightening spirit in the 20th century. In society it will be marginalized even though to this day it is, according to Eric Kandel, the most exciting and complex theory of the human spirit. Although many scientific theoretical and philosophical arguments could be imposed against this point of view, Kandel's assessment is surely correct in the sense that proving the sustainability of psychoanalysis and psychoanalytic therapies with neurobiological tests such as fMRI or EEGs would immediately enhance the acceptance of psychoanalytic procedures within the world of medicine.

Keeping this in mind, we perceived the opportunity of an institutional cooperation with the Max Planck Institute for Brain Research in Frankfurt, a.M. as an enormous chance to additionally examine a number of chronically depressed patients in our LAC study^3^ with fMRI and EEGs (at the sleep laboratory of the SFI) which is designed as a replication of the Hanse-Neuropsychoanalysis Study (see Buchheim et al., 2012). The already tested methods of the Hanse-Neuropsychoanalysis Study are implemented here in combination with our sleep-dream-research. This is an on-going study, the so-called FRED Study (see below). Therefore, at the moment, we can only give an account of our attempts to combine *psychoanalytic* and *neuroscientific methods* within this study by presenting a single case study, which will be illustrated in the following section.

In the last sequences of this paper we also would like to illustrate our attempt to combine *clinical* and *extra-clinical (experimental) research* in the LAC depression study in another single case study. Marianne Leuzinger-Bohleber has reported the changes of dreams of a severely traumatized, chronic depressed patient as one indicator for therapeutic changes from a clinical perspective in another paper (Leuzinger-Bohleber, 2012). The same patient, part of a subsample of the 426 chronic depressed patients recruited in the LAC depression study, was willing to spend the necessary nights in the sleep laboratory of the Sigmund-Freud-Institute since investigating his severe sleeping disturbances was of clinical importance. The patient's thus elicited EEG data indeed showed pathological sleep patterns so that he had to be referred to a medical expert for sleep disturbances. As a “by-product” of this “therapeutic intervention” in the sleep laboratory, we were able to evaluate and compare his dreams obtained in the laboratory with those reported in psychoanalysis by two researchers independently. The dreams obtained in psychoanalytic treatment were analyzed and evaluated by the clinician in the psychoanalytical treatment as the analysand spontaneously reported them (see p. 19) and all dreams collected in the laboratory setting after REM awakenings were analyzed with the Moser method^4^ (see p. 20) quantifying occurring changes in a standardized evaluation^5^. As laboratory dreams and home/clinical dreams are not different in a descriptive manner (length, word-count, narrativity) a comparison of both types of dreams seemed plausible. What makes them different is to whom a dream is told—a total stranger or a clinician with whom you have a close relationship. This difference shows itself with respect to dream elements told, where home dreams contain significantly more sexual and aggression/misfortune elements and laboratory dreams more bizarre elements (Foulkes, 1979; Schredl and Wittmann, 2005). The aim here was to test if changes found in clinical dreams (dream type A) with a psychoanalytic evaluation (method 1) can be found in laboratory dreams (dream type B) with the Moser method (method 2) as well, which would be indicative for a great robustness of the effect (changes in dreams).

## The frankfurt fMRI/EEG depression study- FRED: approaching psychoanalytic transformation processes in traumatized, chronically depressed patients with the help of imaging procedures^6^

FRED^7^ (Frankfurt fMRI/EEG Depression Study) is an example of a fruitful combination of two domains—psychoanalysis and neurosciences. This very ambitious project currently conducted at the Sigmund-Freud-Institut (SFI) and BIC (Brain Imaging Center) in cooperation with the MPIH Frankfurt (Max-Planck-Institute for brain research)^8^ seeks to examine changes of brain functions in chronic depressed patients after long-term therapies aiming to find multi-modal-neurobiological changes in the course of psychotherapies.

When looking at depression from the angle of brain-physiology, some interesting findings have been put forth: for instance that depression is related to a neurotransmitter disorder, or a frontal lobe dysfunction (cf. Caspi et al., 2003; Belmaker and Agam, 2008; Risch et al., 2009). Northoff and Hayes (2011) have convincingly put forth the theory that the so-called “reward system” is disturbed in depression and that there is evidence that deep brain-stimulation can improve severe depression (also see Solms and Panksepp, 2012).

But despite all these findings, no specific brain-physiological marker for depression has yet been found. It is therefore justified to address the research question of whether changes in the course of therapy have brain-physiological correlates, which we are currently investigating in FRED.

Generally speaking, psychotherapists—especially psychoanalysts—work with what can be remembered and with recurring—usually dysfunctional—behaviors and experiences. The assumption is that this has precipitation within the brain, like synapse configuration, priming, axonal budding and more, giving ground to the hypotheses of FRED. This constitutes the neuro-psychoanalytic aspect of the FRED-study of which some preliminary results will be given in the following. Another aspect of change relevant for the study is that of clinical change found in dreams in the course of psychotherapy. The analysis of dreams with the specific method of Moser and von Zeppelin (1996)—as will be outlined—enables the comparison of empirically elicited findings with clinically reported ones from the therapist.

## Methods

### Design of the FRED-fMRI-study

The FRED-Study investigates the hypotheses that (1) psychotherapy is a process of change in encoding conditions of memory and (2) change in memory encoding will precipitate change in brain activation patterns detectable in fMRI scanning. We hypothesized that changes in memory processing during the psychotherapy will impact the processing of trauma related memories. In the FRED study we aimed at highlighting changes in memory processing during the psychotherapy scanning depressed patients during a recognition task involving stimuli related to an underlying conflict, at the beginning of the psychotherapy and 7 and 15 months later. With such a paradigm, we predicted that the contrast [recognition of trauma-related words/sentences vs. control conditions] will highlight brain regions known to be involved in processing self-relatedness and the retrieval of autobiographical memory and/or emotional memory (emotional memory; amygdala, hippocampus, prefrontal cortex, see Buchanan, 2007; episodic memory and processing self-relatedness: medial prefrontal cortex, parietal cortex, temporal poles, see Legrand and Ruby, 2009; autobiographical memory: medial frontal cortex and hippocampus, see Maguire, 2001) and that such a pattern of activation will change across time and in the course of psychotherapy^9^. Our predictions for the session effects are as follows: Healthy control subjects without any treatment show no significant session effects, and the activation patterns remain constant over time. In successfully treated psychotherapy subjects, the patterns of activation are changing from Time 1 to Time 3, therefore producing significant session effects in statistical terms.

**Figure 2:**
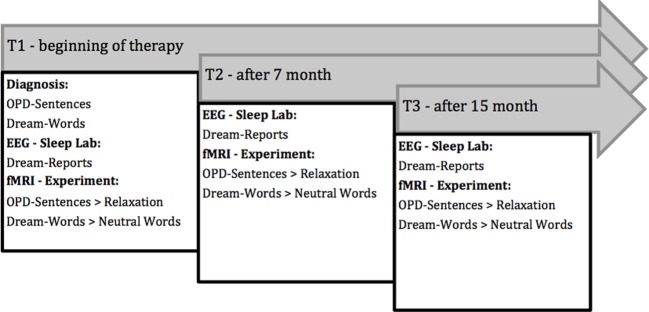
**FRED-Design**.

For this investigation, chronically depressed patients were recruited with whom an Operationalized-Psychodynamic-Diagnostics-Interview (OPD-Interview; OPD-Task Force, 2008) concentrating on axis II (relational) and a dream-interview (see Figure 2 below) were conducted in a first diagnostic phase. From these two interviews the stimuli for the fMRI-scanning are created individually for each patient because they are considered to be good triggers to elicit memory of an underlying conflict. Dream-Words are taken from a significant dream elicited in the dream interview and dysfunctional sentences taken from the OPD-Interview are formulated. Measurements are taken at three different time points revealing changes in activation-patterns occurring during the *course of therapy.* At T1 OPD-Sentences and Dream-Words were elicited and patients spent two nights in the sleep laboratory where verbal Dream-Reports were collected^10^ in the second night after awakenings from REM2^11^ to REM3^12^ and in the morning. Finally the fMRI-Experiment was conducted using the OPD-Sentences and Dream-Words collected previously. At T2 and T3 EEG—Sleep Lab data and fMRI data were collected in the same manner using OPD-Sentences and Dream-Words from T1.

### Participants

At present 16 patients with recurrent major depressive disorders (Major Depression, Dysthymia, Double Depression for more than 24 months; Quick Inventory of Depressive Symptoms (QIDS) > 9 [scale range 0–27, clinical cut-off ≥ 6]; Beck Depression Inventory (BDI) > 17 [scale range 0–63, clinical cut-off: ≥ 9]; age: *M* = 43, range 23–58 years, *SD* = 11.57) take part in the FRED study. Patients of the FRED study were recruited at the Sigmund-Freud-Institut's outpatients department from the LAC-Depression Study (Leuzinger-Bohleber, 2013) conducted there, diagnosed by trained clinicians using the Structured Clinical Interviews I and II for DMS-IV Diagnosis (German version; 1998). Exclusion criteria were other psychiatric conditions as main diagnosis, substance abuse, significant medical or neurological conditions (including medical causes of depression), psychotropic medication, and eye problems. All participants were right-handed. In both groups, depression severity and general symptoms of psychopathology were assessed using the Beck Depression Inventory (BDI, Hautzinger et al., 2006[1995]) and the revised Symptom Check List (SCL-90-R, Franke and Derogatis, 2002), respectively. The control-group (13 females) consists of 18 healthy volunteers matched in age (*M* = 34, range 22–65 years, *SD* = 14.59). All participants gave written informed consent.

### Stimuli

#### Dream—stimulus

To gather individualized and personally relevant stimuli relating to dreams dream interviews were performed with each subject eliciting a significant (recurrent) dream of which 30 Dream-Words were extracted together with the subject, paying close attention that they reflect the narrated dreams as concisely as possible and as close to the dream experience as possible. The dream interviews were conducted by a trained clinician (TF) and audiotaped. The participants were asked to memorize these words 1 day prior to the fMRI-investigation. These 30 Dream-Words served as stimuli during the fMRI-session (dream experiment). The control condition comprised 30 accordant words taken out of a subjectively neutral “everyday life-story,” which had no specific meaning for the individual patient^13^ and was taken out from a travel report in a newspaper article describing a camping vacation. They were matched in length and frequency of the words in the native language of the patient (Neutral-Words). The participant was instructed to memorize these words as well 1 day prior to fMRI-scanning. These 30 Neutral-Words served as stimuli during the fMRI-session (neutral condition). All words were presented in German.

#### OPD-stimulus

**Figure 3:**
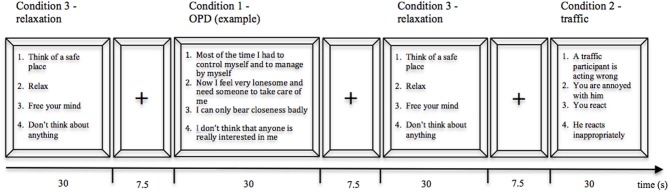
**Block-design for OPD stimulus presentation.** Each of the 4 sentences of a condition was presented for 7.5 s in a fixed order 1–4.

The individualized and personally relevant stimuli relating to depressive symptoms were extracted from an OPD interview (Operationalized Psychodynamic Diagnosis; OPD-Task-Force, 2008), which were conducted with each patient. OPD is a multiaxial system assessing psychopathology focusing on pure description of symptoms (Axis V), experience of illness (Axis I), dysfunctional interpersonal relations (Axis II), psychodynamic conflicts (Axis III), and psychological structure (Axis IV) (OPD-Task-Force, 2008). The OPD interviews were conducted by trained clinicians and the dysfunctional relations blind rated independently by 2 experts. From the systematic and item-based diagnosis (OPD-Task-Force, 2008), four sentences were identified representing the core dysfunctional relationship theme of each participant (cf. Figure 3; condition 1).

The control condition comprised four statements of a stressful traffic situation to induce negative emotions and recall autobiographical memories with a personally relevant situation including human interactions, but without engaging in material that might interfere with participants' depression or interpersonal distress (cf. Figure 3; condition 2).

To allow participants to recover after emotionally demanding sentences, “relaxation” sentences were inserted between the OPD- and control condition. These sentences instructed participants to relax by thinking of a safe place. Subjects were prepared for the “relaxation” condition before the experiment (cf. Figure 3; condition 3).

Whereas the OPD sentences were derived individually for each person, “relaxation” and “control” were the same sentences across all subjects. All sentences were presented in German.

### Procedure

**Figure 4:**
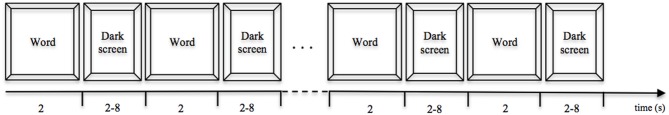
**Event-related design for Dream stimulus presentation.** Word series of 120 words comprising 30 Dream-Words and 30 Neutral-Words previously learned +60 Distractor-Words (not learned); presented in random order with variable Inter-Stimulus-Interval (ISI) of 2–8 s (mean 6.2 s).

The fMRI dream-word experiment was run as a within-subjects design with a learning/encoding phase, and a retrieval phase (during fMRI measurements). In the learning phase subjects memorized 30 Dream-Words (from his significant dream reported earlier) and 30 Neutral-Words (from a non-individual everyday life story), up to 2 days prior to fMRI scanning. At the beginning of the fMRI session and prior to scanning, subjects were presented with their individual Dream-Words as well as with their individual OPD sentences and asked whether these words adequately represented his significant dream and whether these sentences adequately represented their problematic relations. Each word series comprised 60 randomized words consisting of previously memorized 30 Dream-Words and previously memorized 30 Neutral-Words each. During recognition, subjects were presented with the 60 target words, mixed with an additional 60 Distractor-Words. The whole series of 120 items was randomized for each subject. After scanning a questionnaire assessing on a 7-point Likert scale, the extent to which the presented 120 words caused emotional arousal was given. Lying in the scanner, subjects were tested for recognition of the words learned previously. Items were presented during the recognition test for 2 s with a variable Inter-Stimulus-Interval (ISI) at a mean rate of 6.2 s randomly jumping between 2 and 8 s. Subjects had to decide on each of the 120 items, if it was old or new, by pressing one of two pre-assigned buttons with the thumb of the right hand, which was stated as being the dominant hand by all subjects. During the subsequent anatomic measurement (MPRAGE) the subjects where surveyed for their current affectivity in the scanner. Dichotomous items of the affectivity scale (Befindlichkeitsskala-Bf-S, von Zerssen, 1976, German) were presented by pairs (e.g., restful-restless) and the question asked: “Which word rather applies?” The answer was given by pressing one of two response keys (left-right). The dream experiment lasted approximately 30 min (cf. Figure 4).

For the OPD—experiment (block design) the four sentences of each condition (OPD, control, relaxation) were individually presented for 7.5 s while in the scanner. During the OPD block participants were asked to mentally engage in situations with significant others described by the OPD sentences and further instructed to allow spontaneous thoughts, emotions and memories to come to mind. The “control” and “relaxation” conditions also included four sentences each lasting 7.5 s. The instructions were to mentally engage either in the recalled traffic situation or to relax. The 12 “relaxation”, six “control,” and six “OPD” blocks were separated by a 7.5-s fixation cross. The OPD experiment lasted approximately 15 min (cf. Figure 3).

On retest at time points T2 and T3, the forgetting and blurring factor was considered by means of a memory refreshing procedure before scanning. Subjects were shown again the 30 dream-words as a list and asked to remember and memorize their dreams related to these words. The 30 words from the neutral story were presented as well, and the related text was read again.

### Measurement

fMRI was performed using a 3.0-Tesla head-scanner (Magnetom Allegra, Siemens, Erlangen, Germany) with a 4-channel-head coil applying an EPI mosaic sequence (*FA* = 90°, *TE* = 30 ms, matrix 64 × 64, interleaved acquisition, voxel size 3 × 3 × 3 mm, 1.5 mm gap, 30 transverse slices covering the whole brain, T → C = −30°), obtaining a series (370 measurements) of blood-oxygenation-sensitive echoplanar image volumes every 2 s.

### Data analysis

The functional data were analyzed using the SPM 8 software from the Wellcome Department of Cognitive Neurology, London, UK, running under Matlab 12b (Mathworks Inc., Sherborn, MA). All images were realigned (for motion correction, slice timing correction), normalized into a standard space (MNI template, Montreal Neurological Institute), and smoothed with an 8-mm full-width-at-half-maximum Gaussian kernel. For the dream-experiment a within-subject model (first level) was calculated with six conditions (Dream right and false, Neutral right and false, Distractor right and false) and three sessions (T1, T2, T3). False-conditions were variables of non-interest, contrasts Dream > Neutral (correct responses only) in T1–T3 were the effects of interest. The significance of the session-effect from T1 to T3 was estimated. The OPD-experiment was analyzed as a block-design with three conditions (OPD, Traffic, Relaxation) and three sessions (T1, T2, T3), contrast OPD > Relaxation and the session-effect T1/T2/T3 were calculated. In both experiments the head movement parameters were included as covariates of non-interest (ANCOVA without grand mean scaling).

## Results

Since the study is still on-going and reliable results of the group analysis are not yet obtained, results of a single case analysis (Mrs A., female, 50 years of age) is presented here for illustration purposes. The patient underwent a 3-year psychoanalytic treatment during which she was tested three times in the fMRI. With respect to the behavioral data, her own perception and according to the questionnaire parameters, depressive symptoms improved significantly during this period (BDI recruitment/first year/third year: 24/15/11; QIDS-C: 9/4/2).

### Behavioral data

Analyses of the percentage of correctly recognized target items revealed a high rate of correct responses for Dream-Words (30/30/30), Neutral-Words (28/29/29), and Distractors (58/58/59 out of 60) in the three sessions (T1/T2/T3). Mean reaction time for Dream-Words was 833 ms, for Neutral-Words 848 ms, and for Distractors 995 ms. Dream-Words produced more negative emotional arousal (T1-Rating = −3.2) than Neutral-Words (+0.2) and Distractor words (+0.1). The affectivity distinctly improved from T1 to T3 (Bf-S = 48/36/29).

### fMRI data

**Figure 5:**
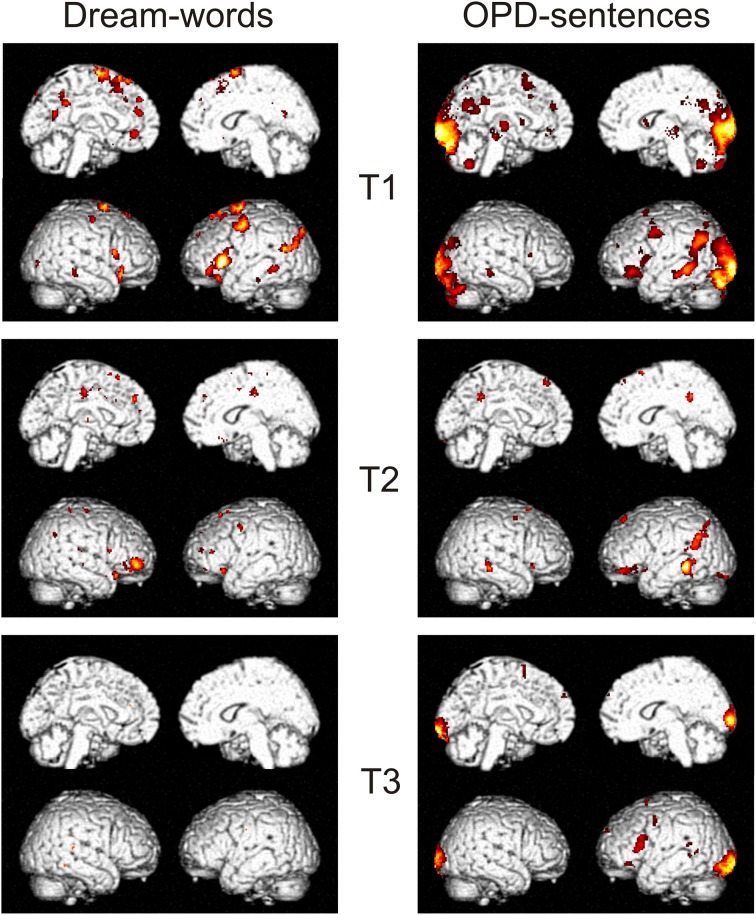
**T-contrasts Dream words > Neutral words (left side) and OPD-sentences > Relaxation (right) of a single subject (Subject Mrs A.) over time T1–T3, all thresholds to *p* < 0.05, *T* > 3.2, 10 voxels minimum, and corrected for multiple comparisons (FDR)**.

Results of the dream-experiment revealed, that Dream-Words in contrast to Neutral-Words showed a differential activation. The contrast Dream > Neutral was significant in T1 and T2, but not anymore in T3 (see Figure 5). The contrast showed at T1 a widespread activation pattern in left inferior frontal (area 44 and 45), left superior frontal (area 6), left intra parietal cortex (angular gyrus, middle temporal gyrus), left middle occipital gyrus, right inferior frontal gyrus pars orbitalis, right temporal pole, left precuneus, left anterior cingulate cortex, left superior medial gyrus, right inferior frontal gyrus/pars orbitalis, right middle temporal gyrus, left superior medial gyrus, left medial frontal gyrus. From T1 to T2 this distinct pattern—being significant to emotional processing of the self—largely disappears. The complete disappearance of the pattern at T3 alludes to the assumption that the dream content has lost its special importance and is now experienced in the same manner as the neutral story (cf. Table 1). The calculated session-effect was significant (*F* = 6.8, *p* < 0.05 FDR, corrected for multiple comparisons).

**Table 1:** **Contrast Dream-Words > Neutral-Words at time point T1**.

**Anatomical region**	**Hemisphere**	**BA**	**MNI**			**Voxel**	***T***
			***x***	***y***	***z***		
Infer frontal gyrus	Left	44/45/3b	−48	18	4	833	6.7
Superior frontal gyrus	Left	6	−16	−2	72	683	6.0
Postcentral gyrus	Left	6	−50	−8	52	397	6.1
Intraparietal cortex (PGp, PGa, PFm)	Left	39/40	−46	−74	26	370	4.5
Superior frontal gyrus	Right	6	14	4	74	171	6.5
Inferior frontal gyrus (orbital)	Right	45	48	26	−8	131	4.6
Anterior cingulate cortex	Left	32	−12	42	−4	89	5.0
Middle temporal gyrus	Left	35/36	−54	−44	−4	88	4.6
Superior medial gyrus	Left	32	−10	48	20	80	4.0
Inferior frontal gyrus (orbital)	Right	45	34	22	−14	71	3.8
Middle temporal gyrus	Right	22	50	−34	−8	49	4.1
Precentral gyrus	Right	6	54	−12	60	44	4.6
Superior medial gyrus	Left	32	−6	48	38	44	3.2
Middle frontal gyrus	Left	8	−28	16	64	40	3.8

All clusters significant after correction for multiple comparisons (FDR, p < 0.05).

MNI xyz, local maxima coordinates (Montreal Neurological Institute), T-test statistics, BA-Brodmann area.

In the OPD-experiment the contrast OPD-sentences > Relaxation was significant at T1–T3 as well. The widespread activation pattern at T1 occipital (area 17 and 18), left and right hippocampus, left and right thalamus, left precuneus, left middle cingulated cortex, left inferior frontal gyrus/pars orbitalis, left precentral gyrus, vermis, left inferior frontal gyrus, right middle temporal gyrus, superior parietal lobule, cerebellum, and right and left putamen was markedly reduced at T2 and T3 (cf. Table 2). The calculated session-effect for this contrast was significant (*F* = 10.3, *p* < 0.05 FWE, corrected for multiple comparisons).

**Table 2:** **Contrast OPD-sentences > Relaxation at time point T1**.

**Anatomical region**	**Hemisphere**	**MNI**			**Voxel**	***T***
		***x***	***y***	***z***		
1. Occipital lobe/Cerebellum/Lingual gyrus/Fusiform gyrus	Left/Right	(Multiple clusters)			12,590	>2.7
2. Thalamus/Hippocampus:	Left				464	>2.7
Thalamus	Left	−12	−14	6		3.6
		−14	−16	4		3.5
Hippocampus	Left	−24	−26	−10		4.4
3. Precuneus/Cingulate:	Left				421	>2.7
Precuneus	Left	0	−62	34		3.5
		−4	−56	22		3.0
Middle cingulate	Left	−4	−38	36		3.2
		−8	−42	38		3.1
4. Inferior frontal gyrus	Left				339	>2.7
		−50	34	−12		3.4
		−38	32	−4		3.8
		−46	24	−10		3.6
5. Hippocampus/Thalamus	Right				295	>2.7
Thalamus	Right	26	−26	−4		4.8
Hippocampus	Right	28	−22	−16		4.4
		28	−22	−16		2.9
6. Precentral gyrus	Left				273	>2.7
		−46	2	40		4.1
		−50	0	48		3.3
		−36	−2	46		3.0
7. Inferior frontal gyrus	Left	−44	10	10	98	3.3
8. Middle temproal gyrus	Right	58	−32	−10	67	3.5
9. Superior parietal gyrus	Left	−26	−66	54	50	3.2
10. Superior frontal gyrus	Left	−12	46	30	48	3.3
11. Putamen	Right	20	10	6	39	3.3
12. Putamen	Left	−20	18	44	32	3.2

All clusters significant after correction for multiple comparisons (FDR, p < 0.05).

MNI xyz, local maxima coordinates (Montreal Neurological Institute), T-test statistics.

Both experiments distinctly showed a marked decrease of activation, lower pattern differentiation and even partial disappearance of patterns in the course of time from T1 to T3. In order to know whether or not the evolution of this pattern of activation from T1 to T3 is related to psychotherapy, it is needed to compare patients' data with control subjects' data. The data found thus far do not contradict this notion.

## Psychoanalytic evaluation of changes in dreams during treatment

Within the FRED-study, dreams were not only studied from a neurophysiological perspective but also from a psychoanalytic standpoint by analysing the manifest dream using the Moser method. This method is based on the analysis of dreams under problem-solving aspects, which strongly relies on affect-regulation, since the success or failure to resolve a conflictuous complex, assumed to be underlying the dream, will ultimately be determined by it (see below). Analysis is done by scrutinizing the manifest dreams for certain aspects, among others: elements positioned within the dream-world, observable interactions taking place between self and others or the absence of them and interruption of dream-scenes, which allude to affective overflow rendering such interrupts necessary. The following describes the dream coding system of Moser and von Zeppelin as it has been applied here.

## The dream coding system of moser and von zeppelin

Based on their model of cognitive-affect regulation, Moser and von Zeppelin (1996) have developed a coding system which can be used to analyse dream material. It is an evaluation system with formal criteria to investigate manifest dream-content and its changing structures (for a more detailed description cf. Fischmann et al., 2012a).

According to Moser and von Zeppelin the regulating processes of dream-organization as described in our introductory remarks can be detected by:

- how elements are positioned in the dream-world (i.e., potentiality for involvement)
- monitoring the dream activity (preparing or omitting involvement),
- allocating affective feedback information of each dream-situation and its consequences,
- allocating regulating procedures responsible for changes (interaction).

**Figure 6:**
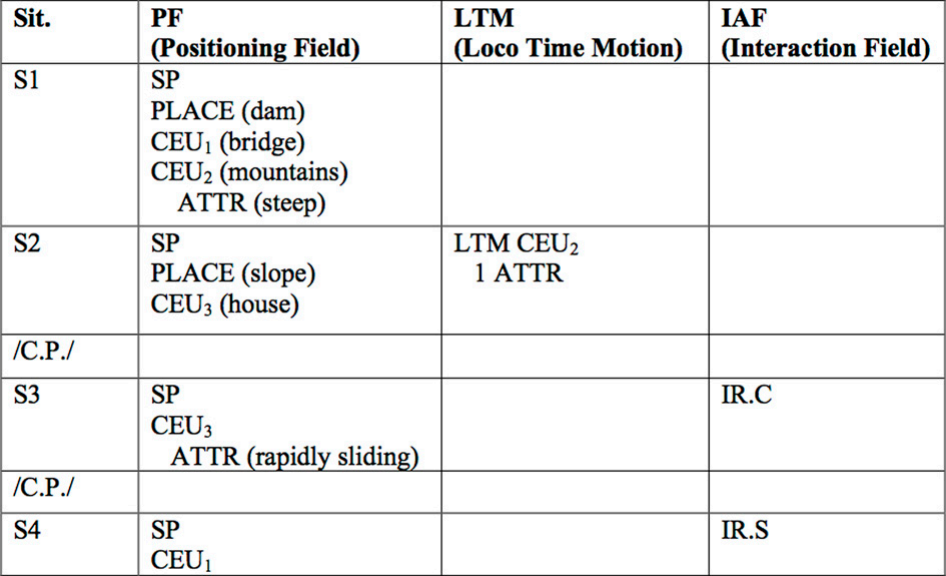
**Moser and von Zeppelin dream coding sheet.** SNbr, Situation (dream-scene); /C.P./, cognitive process (Interrupt); SP, self processor (dreamer); CEU, cognitive element inanimate; ATTR, attribute; IR.C, interactive relation connecting; IR.S, interactive relation self-changing.

The coding system defines formal criteria and structures of a dream discernable within the manifest dream narrative elucidating affect-regulation processes of the dream (see Figure 6): number of situations, type of places and social settings named in a dream (descriptions, attributes), objects occurring (descriptions, attributes), placement, movement, interactions of objects as well as the question of whether the dreamer himself was involved in interactions, or if he remains a spectator.

As mentioned above two principles of affect-regulation are assumed: (A) the security-principle and (B) the involvement-principle, which can be discriminated by the “positioning” of elements within the dream and through “interaction.” Common to both principles is their ruling by negative and positive affects, i.e., anxiety is the motor for an enlargement of security also regulating involvement by, for instance, breaking off interaction and generating a new situation. It is assumed that problem solving can only take place and be tested in interaction; therefore the dream tends toward interaction.

It is assumed that the more elements used in a dream scene, the more possibilities are available for the dreamer to regulate his affects and contents processed in the dream. If the dream omits “interaction,” security aspects are dominant.

The following clinical case focuses on the analyses of two distinct dream series from the first 3 years of therapy. For one a dream series enfolding two dreams taken from clinical sessions of the psychoanalytic treatment (clinical dreams)^14^ is compared to a dream series comprising four dreams, which were elicited in the dream laboratory (laboratory dreams). This comparison highlights how clinical and experimental data combined give mutually enriching insights into changes occurring during the course of treatment.

## Clinical case: analysis of a dream series in the course of therapy

### Biography and trauma history

Marianne Leuzinger-Bohleber has described the clinical and biographical background of this severely traumatized, chronic depressed patient extensively in another paper (Leuzinger-Bohleber, 2012). There she illustrated from her clinical perspective how the manifest dreams as well as the dream work changed during psychoanalysis and also reported on the transformation of the inner (traumatic) object world. In this paper we would like to contrast her clinical views with a more systematic investigation of the changes in the manifest dreams.

Here a short summary of the clinical material:

The patient explained in the assessment interviews that he had been suffering from severe depression for the last 25 years, and that he came to our Institute because after the last depressive breakdown he had submitted an application for a retirement pension. The doctor who assessed his application concluded that he did not require a pension, but an “intelligent psychoanalysis,”—initially a response Mr W. found highly insulting. He felt that he had not been taken seriously, especially his substantial physical symptoms; the unbearable pains involving his entire body, his acute eating disorders as well as his suicidal tendencies. Furthermore, the patient suffered under severe sleeping disorders. Often he is unable to sleep at all. As a rule, he wakes up after one and a half hours, or after 3 h at the most. He feels physically exhausted and is barely able to concentrate on anything.

Mr W. had already undergone several unsuccessful attempts at therapy, including behavioral therapy, Gestalt Therapy, “body therapy” as well as several inpatient treatments in psychiatric and psychosomatic clinics. He is among the group of patients that for the most part seem unable to respond to psychotropic drugs, and whose relapses occur at ever-shorter intervals and with increasing intensity. After many consultations with various psychiatrists and neurologists, he then discovered that solely Lyrica^15^ enabled him more or less to deal with his states of physical stress and his anxiety attacks.

The patient is an only child. One of the known details about his early history is that he was a “cry-baby.” When he was 4 years old Mr W.'s mother fell seriously ill. W. was admitted to a convalescent home for children, evidently founded on authoritarian, inhumane educational principles reminiscent of National Socialist ethos. Just how traumatic an experience this stay in a home was, is something that became apparent during psychoanalysis. Mr W.'s first childhood memories revolve around the following event: he recalls how his father took him by the hand and led him out of the home. He also recalls how a girl had been forced to eat her own vomit.

Mr W. experienced two further separations from his ill mother, but these incidents had proven less traumatic since he had been taken in by relatives.

In spite of the dissociative states following the traumatic separations and his social isolation, W. was a good pupil, who went on to complete his first apprenticeship training and later his university studies. During adolescence, he had a psychosomatic breakdown, which the parents diagnosed as a “growing up crisis.” At the age of 15 years, he met his first girlfriend. His condition improved. At the age of 22 he ended the relationship with his first girlfriend because he fell in love with another woman. Although the separation ran in his favor, he reacted very severely to it. Although he had also initiated the separation from his second girlfriend, he suffered for weeks due to the separation. After entering another relationship he was dramatically overcome by a nervous breakdown during a party held by his new girlfriend: he had to be taken to hospital due to hyper-ventilation (panic attacks).

As already mentioned, Mr W. had undergone several psychotherapies. Although all his therapies alleviated his problems, “none of them cured him.” His depressions became worse and worse until they became chronic.

During the current treatment his self-reported depressive symptomatology improved over the first 2 years of treatment considered here (BDI recruitment/first year/ beginning of the third year: 48/40/30), whereas external assessment of depressive symptomatology by a trained clinician in the LIFE interview remained salient (QIDS-C: 15/16/17). It is interesting that the beginning transformation of the inner world of the patient became observable in the changes of the manifest dreams (see below), but, after the first 2 years of treatment, had not been seen yet by the independent clinician in the LIFE interviews.

### Dream series elicited in the psychoanalytic treatment

Within the framework of this paper we cannot elaborate on the psychoanalytic understanding of the transformations of the manifest dreams or on the work with the dream associations in the psychoanalytic sessions (see e.g., Leuzinger-Bohleber, 2012, 2013). We can only communicate a first impression of these changes in two dreams, one from the end of the first and one from the third year of treatment. The first dream reported here is a typical dream of a severely traumatized person where the patient himself is in a position of an observer: the dream subject is in an extreme, life threatening situation, completely helpless, in unbearable pain—and not being helped by anyone. In the second dream the patient is the active dreamer, observing a situation which still is painful but with hope that “something can be done” in order to overcome a hopeless situation

### Clinical dream 1: first year of treatment

“I catch sight of a man lying at the side of the road severely wounded—his intestines are spewing out, and everything is saturated in blood… A helicopter appears. It is unclear as to whether the man is still being shot at, or whether one should go to his aid. Someone appears claiming that the man now has passed away. I notice that the man is still alive and he really does open his eyes and enquires: why is nobody helping me? The woman hands him a lid of a saucepan, which he should hold over his open wound … I then wake up, riveted by panic… ” (Leuzinger-Bohleber, 2012, p. 66/67)

### Clinical dream 2: beginning of third year of treatment

“I am gazing at a group of people all smeared with clay who are working together on the outer shell of a house. A cold wind blows—the work is torturous, arduous, and barely tolerable. And yet, in the dream I have a certain sense that the men will succeed: at some point the house will be built and provide them with a warm home. I then turn to my wife and say: “You see, we can do it –one just has to stay together… ” (Leuzinger-Bohleber, 2012, p. 70/71)

By comparing the clinical dream from the beginning of psychoanalysis with the one of the beginning of the third year of analysis, Leuzinger-Bohleber observed changes in the patterns of the relationships, where *the dream-subject shows better relationships with others* (e.g., people helping each other in the second reported dream). In the first dream the dream subject had mostly been alone: no one helped him and soothed his anxieties, panic and despair. *The range of actions of the dream-subject is increased* and *the emotional spectrum is enlarged* (in the dreams at the beginning of psychoanalysis we find only panic—at the beginning of the third year of analysis we also observe surprise, joy, satisfaction, humor and yet continuous anxieties and pain).

There is also a noted change in the dream atmosphere, with the variety of affects as well as its increased intensities and manifest anxiety being less frequent. The dreamer's increased capability to perceive different and even contradictory emotions become more and more visible. New feelings of anger, rage but also positive affections, tenderness and sexual attractions appear in the dreams toward the second year of treatment. The dream subject is no longer a (distant) observer but plays an active part and is involved in intensive emotional interaction with others.

Furthermore, Leuzinger-Bohleber distinguished *clearer problem-solving strategies* (more successful than non-successful problem-solving) and a *broader range of different problem-solving strategies* from the manifest dreams. The dream-subject is no longer as flooded as in a traumatic situation in which he experiences extreme helplessness and lack of power. In his dreams around the beginning of the third year of psychoanalysis he encounters objects willing to help and support him. This seems to be a very important indicator that the inner object world of the severely traumatized patient has changed (see introduction and Leuzinger-Bohleber, 2012, 2013).

### Dream series elicited in the dream laboratory

In the following a total of four dreams—two from the end of the first year of therapy and two from the end of the second year all elicited in the dream laboratory—will be analyzed for changes within the course of therapy using the Moser method.

### Laboratory dream 1—end of first year of treatment

“I am standing on a bridge over a dam. To my right and left are steep slopes—mountains (S1). There is a landslide. I see the slope and an entire house approaching me very fast, rapidly sliding rushing toward me (S2). I think to myself, that I will not be able to escape it (/C.P./). I am running (S3) and am amazed at how fast I can run (/C.P./). I succeed in saving myself from the rapidly approaching house (S3). I am in safety at the edge of this bridge (S4).”

In order to analyse this dream with the Moser method each and every element of a situation is given a code (cf. Figure 3) in the respective column of either the positioning field (PF), the field of trajectories (LTM) or the interaction field (IAF):

From here the dream can be analyzed as follows: the first situation of this dream (S1) is coined by the security principle—many cognitive elements are simply being placed. But it also hosts a multitude of involvement potential as many attributes are being named for the elements placed. In the second situation (S2) a first attempt is made to deal with this potential—albeit rather limited (LTM)—but again increasing potentiality by adding another attribute (ATTR). As a result the affectivity seems to increase to such an extent that the dream-scene has to be interrupted by a comment (/C.P./). In S3 the dreamer finally succeeds to invoke a “*successful*” interaction between the threatening cognitive element [CEU_3_ (house)] and himself (SP). Initially this leads to another interrupt: the dreamer is surprised by his capabilities and finally in S4 a cathartic self-changing interaction is conjured up: he is in safety.

In summary the patient describes a threatening situation, which is initially determined by the security-principle. The relatively sophisticated description of the first scene bears potential, which the dreamer makes full use of in order to regulate the threatening affects. The wish to “bring himself to safety” is fulfilled in this dream.

### Laboratory dream 2—end of first year of treatment

“There are more people in the room. I wear this cap. You three are here and somebody else, who will come up right after me. He makes a lot of pretensions. It is morning and I wake up. I wear this cap and am hooked up to all those cables (S1). It is lively around me and you and the others are walking around, talking to each other. I pick up on you whispering and being annoyed at someone or making fun of him. The one that you are annoyed with is in the room as well, and he is supposed to put the cap on after me (S2). I remember that I have seen him once before in front of the door of my analyst (S3). He is here in the room and constantly makes pretensions. Everything should be the way he wants it. You are annoyed that you have to fulfill these wishes (S4). I think to myself: “Just take it easy” (/C.P./).”

Obviously this is a “laboratory dream.” The patient uses the research situation as an opportunity to regulate his anxieties of being “too pretentious.” He projects this onto an object processor (OP) turning into an observer. Thus, he successfully distances himself, which gives him the possibility to comprehend the events in more detail.

In the 1st situation (S1) there is a lot of potential to regulate affects—albeit still governed by the security principle. It includes a social setting (SOC SET), variable attributes (ATTR) and a lot of processors inviting action. By placing another patient (OP_2_) into the dream scene the dreamer (subject processor SP) gets the opportunity to take an observational stance, which leads to a movement (trajectory LTM) of the OP_1_ group of researchers in S2. S3 is regulated by the security principle and the potential existent in S2 (LTM) cannot be exploited in S3. In S4 finally this is achieved by an interaction just to disembogue in another interrupt. The affectivity of the situation increases to such an extent that it has to be interrupted: the dreamer cautions the object processor (OP_2_) or rather himself “to take it easy.”

### Laboratory dream 3—end of second year of treatment

“A Formula-1 race with Michael Schumacher (S1). Directly after the race he flies to Germany, in order to inaugurate a bridge (S2). Totally bonkers (/C.P./). He is in Germany and inaugurates the bridge (S3). He speaks with a few people sitting at a table. I am sitting at the table next to it and observe him and the others in debate (S4). How do I come up with something like this? (/C.P./)”

Again the dreamer takes an observational stance. In contrast to the previous dream he succeeds in creating a connecting interaction between two CEs, which is not interrupted but seamlessly leads into a displacement relation. Although this may still be considered to be a distancing manoeuvre from an affective event, it is not as marked as in the previous dream. The involvement principle is more distinct here than it had been previously. The interrupt at the end of the dream is not a rebuke as before, but rather expresses astonishment at what occupies his mind and a (conscious) approximation to the underlying complex may be assumed.

### Laboratory dream 4—end of second year of treatment

“I am on my way with my little son. Other children and adults are with us. A boy is there too, who has something against my son. It is summer. It is warm. We are walking along the banks of a river (S1). We want to buy a wagon or trailer (S2). The children are of different ages. One boy is already 11 or 12 years old. This boy is on edge, because the other children and also my son are so young and they cannot do what he wants them to do, because they are too small (S3). Then my mother appears. She sews a button back onto my shirt (S4). I don't know how this fits in (/C.P./). I say: “Just leave this stupid button alone.” This unnerves me (S5). I am there to oversee everything. A woman is there too. She is the mother of that boy (S1).

This dream is regulated from the beginning by the involvement principle, which alludes to an advanced therapeutic effect. In all successive situations more interactions appear: also connecting self-changing relations of subjects and objects. The self-processor (SP) himself is involved and does not have to retreat into an observing position anymore (no IR.D)—he faces his affects increasingly. After S4 triggers an interrupt, the dreamer (SP) interactively “fends this off” via verbal relation (V.R.). Thus, we might assume that the dreamer progressively deals with the affects underlying the dream-complex in an interactive manner and is able to depict them in dream scenes. The affects are no longer isolated—which implies that previously isolated affects of the dream-complex can be integrated now.

In summary, the analysis shows that the patient's laboratory dreams from the end of his first year in therapy were abundant with anxieties and yearning for security making him hesitant to get involved with others. But even in these dreams he already showed potential of what we might consider to be the result of the on-going therapy, i.e., signs of involvement abilities, enabling him to make use of others by projecting his fears into them and *testing* if he could bear the rising anxieties involved in the actions he projected onto them while he still remained in a distant observer position. But at this stage of therapy his fears of getting involved got the better of him and he could not yet exploit these potentials. At the end of the second year of analysis his dreams reveal his enhanced abilities to get involved (dream 4 is largely dominated by the involvement principle from the beginning) being abundant with interactions with others portraying his increased ability to face his affects. Albeit rising affectivity is still met with an *interrupt* it is now followed by a dream scene of a different quality: he can fend off his rising anxiety via an *aggressive* response (V.R. S5 in dream 4) heralding a progressive approach to the underlying (unconscious) conflict-laden dream-complex by integrating affects into existing memory networks.

**Figure 7:**
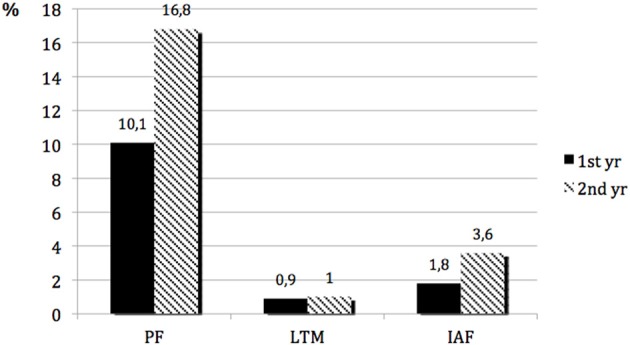
**Relative frequency of single codes relativized by the average number of words**.

To illustrate these changes occurring from a more experimental perspective the following graph might be helpful (see Figure 7):

There is a clearly recognizable increase in potentials (PF) from the end of the first year to the end of the second year dreams that can be exploited for interaction (IAF). The finding of an enhanced ability to get involved can be seen here by simply having a look at the manifest dreams.

## Concluding remarks

This extra-clinical analysis of the manifest dream-content of the patient's laboratory dreams substantiate his clinical improvement as Leuzinger-Bohleber illustrated in her analysis of the transformations of the manifest content of the clinical dreams (see Leuzinger-Bohleber, 2012).

The consistencies of the clinical and extra-clinical analyses are remarkable, which from a scientific perspective is of utmost relevance. But to be sure, the clinical case study still provides greater psychodynamically relevant clinical and structural information, as the extra-clinical analysis suffices with the content of the manifest dreams and has no further biographical data at hand with which results could be enhanced. The consistency in the finding on the other hand consolidates the reliability of the clinical case analysis.

Combining clinical and extra-clinical research remains a great challenge particularly in psychoanalytic psychotherapy research. It is still a strength of clinical research in psychoanalysis to communicate the unique and complex insights gained in intensive psychoanalyses by narratives because many a “truth can only be told and not be measured.” At the same time psychoanalysis, as all “contemporary psychotherapies,” is obliged to show the short-term and long-term effects of their treatments to the psychoanalytic as well as to the non-psychoanalytic community. The latter often requires the consideration of criteria of the so-called evidence based medicine in such effectiveness studies (see political context of the LAC depression study, www.sigmund-freud-institut.de). An alternative, innovative approach to “prove” therapeutic changes in an “objective way” is to investigate patients during their psychoanalyses by instruments like the EEG and the fMRI (if the patients are willing to undergo these procedures).

The changes found in the dream material (clinical and laboratory) of the patient presented here could not be tested neurophysiologically as Mr W. had to be excluded from fMRI because of a physical exclusion criterion (heart operation). Therefore, we exemplified clinical changes that have a specific neurobiological resonance by another case of the FRED study that of Mrs A. Data of change using the dream experiment in this single case revealed in the course of therapy the recognition or rather re-sounding of initially significant dream content at the beginning of therapy specifically activated fronto-medial areas, the Precuneus and the Left Parietal Lobe, which did not substantiate after 1 year of therapy. The disappearance of these areas—being significant to emotional processing of the self—at T3 allude to the assumption that the dream content has lost its special importance and is now experienced in the same manner as the neutral story. These changes in and de-differentiation of activation patterns coincided with clinically found improvement. Whether this is indeed related to psychotherapy needs to be analyzed by a group comparison of patients' data with control subjects' data. This will be the subject of upcoming papers.

In this paper we hope to have illustrated the fascinating similarities between the clinical use of dreams as an indicator for changes in the inner (traumatic) object world in psychoanalyses and the systematic, “scientific” investigation of laboratory dreams by the so-called “Moser-method.” We also could show that such changes are also evident on a neurobiological level. The clinical case report focused on the importance of the psychoanalytic context of dreams, the observation of transference and countertransference reactions, the associations of the patient and the analysand etc. necessary to unravel the unconscious meaning of the dream and thus trying to contribute to the “meaning-finding process” of this severely traumatized patient (Leuzinger-Bohleber, 2012). One great advantage of the psychoanalytic clinical “research” on dreams continues to be the understanding of the meaning of a dream in cooperation with the dreamer, that is the patient. His association, and conscious and unconscious reactions to a dream interpretation still are the criteria in order to evaluate the “truth” of an interpretation (see. e. g. Leuzinger-Bohleber, 1987, 1989, 2008). To make a long story short: the transformation of the unconscious world (like dreams)—and as products of it, the maladaptive emotions, cognitions and behaviors (“symptoms”) of the patient—still remain the final psychoanalytic criteria for a therapeutic “success” based on “true insights” of the patient in his unconscious functioning.

On the other hand this kind of “truth” often remains fuzzy and subjective at least in the eyes of the non-psychoanalytic scientific community. Therefore, we have seized the unique opportunity to analyse changes in the manifest dreams by a theory-driven, precise systematic coding system on the one hand and neurobiological evidence on the other hand. These analyses have a high reliability—and inter-subjectivity—and thus may convince independent observers or even critics. Thus, we hope to have illustrated in this paper that the results of clinical research within the frame of intensive psychoanalytical treatments might be combined with extra-clinical research thus emphasizing their empirical, clinical and neurobiological value for future research.

### Conflict of interest statement

The authors declare that the research was conducted in the absence of any commercial or financial relationships that could be construed as a potential conflict of interest.

## Footnotes

^1^To this day psychoanalytic literature is struggling to achieve an adequate understanding of trauma. Bohleber (2010, p. 21) recently summarized the current state of knowledge as follows: “Psychoanalytic trauma theory have evolved on the basis of two models, one psycho-economic, the other hermeneutic based on object relations theory. To grasp the phenomenology and long-term consequences of trauma, we need both models. The psycho-economic model focuses on excessive arousal and on anxiety that cannot be contained by the psyche and that breaks through the shield against stimuli. The model based on object relations theory concentrates on the collapse of internal object relations and the breakdown of internal communication, which produces an experience of total abandonment, precluding the integration of trauma by narrative means.”

^2^Ulrich Moser and Ilka von Zeppelin are fully trained psychoanalysts who have been engaged in interdisciplinary research for decades. Ulrich Moser was professor for Clinical Psychology at the University of Zurich. Since the 1960s and 70s he has been involved in modeling parts of psychoanalytic theories. By the means of computer simulation he tested the logical and terminological consistency of psychoanalytic theories of defence and the generation of dreams. Based on this basic research on dreams, he developed his own model of the generation of dreaming as well as a coding system for investigating the manifest dreams.

^3^In the on-going large LAC depression-study we are comparing the short- and long-term effects of long-term psychoanalytic and cognitive-behavioral psychotherapies with chronic depressed patients. Up to this point we have recruited around 418 chronic depressed patients in different research centers: Frankfurt a. M., Mainz, Berlin, and Hamburg (participating research team and methods: see www.sigmund-freud-institut.de).

^4^The aim here was to test the robustness of the psychotherapeutic effect on dreams of the patient and not to compare two different methods. Therefore we did not analyse the clinical dreams with the Moser method, but chose to assess dreams of the patient in different settings by two different techniques.

^5^As chronically depressed patients in general have a poor dream recall all obtainable dreams out of REM-sleep were analyzed and are all presented here.

^6^This section is a modification of a chapter published in Fonagy et al. (2012), Fischmann et al. (2012b).

^7^Funded by the Neuro-Psychoanalysis Society—HOPE (M. Solms, J. Panksepp et al.) and the Research Advisory Board of the International Psychoanalytic Association.

^8^We are grateful to the BIC and MPIH (W. Singer, A. Stirn, M. Russ) and the Hanse-Neuro-Psychoanalysis-Study (A. Buchheim, H. Kächele, G. Roth, M. Cierpka et al.) and LAC-Depression Study for supporting us in an outstanding way.

^9^Unfortunately, this expected change may also be caused by simple forgetting and “blurring,” not solely due to an effect of psychotherapeutic interventions. This is especially true for the dream-word experiment. Therefore, a control group is needed to observe the “normal” time course in non-treated subjects. Above that, the experimental procedure should take into account forgetting and blurring in the follow-up sessions by appropriate subject instructions (see below).

^10^After 10 min of REM sleep, the dreamer was awakened and asked: “Can you report a dream?”

^11^Second REM sleep period of the night.

^12^Third REM sleep period of the night.

^13^Secured by questioning the subject prior to fMRI-assessment.

^14^The clinical dreams are analyzed by a clinician (M. Leuzinger-Bohleber) using a specific technique largely based on an earlier version of Moser's memory- and affect-regulation-models is applied to the manifest content of the clinical dreams (cf. Leuzinger-Bohleber, 1987, 1989, 2012, p. 324), whereas the laboratory dreams are analyzed by the Moser coding method as outlined above.

^15^Lyrica (generic name: Pregabalin) is an anticonvulsant drug used for neurotic pain, also effective for generalized anxiety disorder (since 2007 approved for this use in the European Union).
